# Effect of CYP3A5 genotypes on pharmacokinetic of tacrolimus in colombian liver transplant patients

**DOI:** 10.3389/fphar.2025.1505490

**Published:** 2025-03-11

**Authors:** Erica Fernanda Lindarte, Gonzalo De Jesus Vasquez, Luis Guillermo Toro Rendón, Andrés Felipe Zuluaga Salazar, Jefferson Antonio Buendia

**Affiliations:** ^1^ Department of Pharmacology and Toxicology, Research Group in Pharmacology and Toxicology “INFARTO”, University of Antioquia, Medellin, Colombia; ^2^ Laboratorio Integrado de Medicina Especializada (LIME), Facultad de Medicina, IPS Universitaria, Universidad de Antioquia, Antioquia, Colombia; ^3^ Grupo de Investigation en Genetica Medica, University of Antioquia, Medellin, Colombia; ^4^ Unidad de Trasplantes y Enfermedades Digestivas, Hospital San Vicente Fundación, Rionegro, Colombia

**Keywords:** tacrolimus, CYP3A5 polymorphisms, liver transplant, Colombia, pharmacogenenomics and personalised medicine

## Abstract

**Background:**

Previous studies have reported a reduced tacrolimus dose-adjusted exposure in individuals expressing the CYP3A5*1 allele (rs 776746). However, information regarding Colombian liver transplantation patients is scarce. This study aimed to investigate the influence of CYP3A5 polymorphism on tacrolimus (TAC) pharmacokinetics in Colombian liver transplant patients.

**Methods:**

This was a prospective, single-center, open-label, pharmacogenetic study in stable adult liver transplant recipients followed up between 2020 and 2022. To evaluate the longitudinal relationship between the Co/doses, dose, and Co and CYP3A5 polymorphisms, a generalized estimating equations model was used using a log-gamma distribution.

**Results:**

We evaluated 16 patients who received TAC during the first 2 years after transplantation. CYP3A5*1 expression was observed 28% of patients. Patients with CYP3A5 expressors displayed lower C0 and C0/dose ratio and higher doses than those no expressors. We observed a lower C0/dose ratio in expresser recipients over 2 years of follow-up.

**Conclusion:**

The expression of CYP3A5 in stable liver transplant patient appeared to have the greatest influence on tacrolimus pharmacokinetics over the first 2 years posttransplant.

## Introduction

Tacrolimus, an immunosuppressive agent, exhibits a narrow therapeutic range and significant interindividual variability in its pharmacokinetics, posing challenges in determining an empirical dosing regimen for organ transplant recipients. Among the potential contributors to this considerable variability, are the genetic association between the CYP3A5 genotype and the pharmacokinetics of tacrolimus (TAC) (Fung, 2023). Specifically, a single nucleotide polymorphism (SNP) located within intron 3 of CYP3A5, denoted as 6986G>A or the CYP3A53 allele, has been shown to exert influence on the pharmacokinetic profile of tacrolimus (Liu et al., 2022). Notably, individuals who are heterozygous or homozygous carriers of the CYP3A51 wild-type allele manifest elevated levels of full-length CYP3A5 messenger RNA and demonstrate heightened expression of functional CYP3A5 protein, herein referred to as CYP3A5 expressers (Hendijani et al., 2018).

Prior investigations have documented an increased demand for tacrolimus dosing and concurrently lower dose-adjusted trough concentrations (C0/Dose) among individuals who are heterozygous or homozygous carriers of the CYP3A51 wild-type allele, in comparison to those with homozygosity for the CYP3A53 variant allele (Lemaitre et al., 2020; Buendia et al., 2015; Buendia et al., 2014). To date, limited research has been conducted on Latin American patients, and no studies have been conducted within the Colombian population (Buendia et al., 2015; Riva et al., 2018; Buendia et al., 2020; Riva et al., 2019; Trezeguet et al., 2023). Given the escalating rates of solid organ transplantations in this geographical region, it has become imperative to augment the body of evidence about the pharmacogenetics of immunosuppressive agents (Diaz et al., 2022). The principal aim of this investigation was to assess the impact of hepatic CYP3A5 genotypes on the pharmacokinetic characteristics of tacrolimus (TAC).

## Methods

This was a prospective, single-center, open-label, pharmacogenetics study in stable liver adult transplant recipients. All recipients provided their written informed consent for genetic analysis. This study was approved by the ethics committee of Hospital San Vicente Fundación Acta No 13-2020. Informed consent was obtained according to institutional guidelines under the Declaration of Helsinki.

### Patients

Patients were recruited within the cohort of liver transplant recipients and followed up between 2020 and 2022. To be included, patients were to be at least 18 years old with a stable dose of tacrolimus administered for no less than 6 months, primary liver transplant recipients with complete or reduced engraftment, stable graft function (aspartate aminotransferase, alanine aminotransferase ≤3 times the reference, alkaline phosphatase ≤5 times the reference value, bilirubin <3 mg/dL), no biliary complications or other surgical complications in the 6 months before the study, no history of rejection in the 6 months before the survey, immunosuppressive therapy with tacrolimus with or without steroids and with or without mycophenolate mofetil, willingness to participate in the study, ability to sign an informed consent. We exclude recipients of a multi-organ transplant or more than one liver graft, human immunodeficiency virus infection, diagnosis of post-transplant cancer, except for patients who had received treatment for basal cell carcinoma, serum creatinine levels >3 mg/Dl, pregnant or breast-feeding women, and women of childbearing age instructed to use contraceptive methods. After liver transplantation, tacrolimus dosing was adjusted to reach a C_0_ between 10 and 15 ng/mL within the first 3 months, between 8 and 12 ng/mL within the first year, and subsequently between 5 and 7 ng/mL. Nevertheless, the daily tacrolimus dose was adjusted according to the clinical state of the patient, especially in cases of toxicity. Patients long-term treated with drugs that are known to interfere with tacrolimus disposition (absorption, distribution, metabolism, and elimination) were excluded.

### Blood sampling and pharmacokinetic analysis

Blood samples were collected just before the morning dose of TAC (C_0_). Tacrolimus blood concentration was measured using an immunoassay, ACMIA (Dimension, SIEMENS) according to the manufacturer’s instructions. Assay calibration was established using calibrators at 0, 3, 6, 12, 20, and 30 ng/mL tested in duplicate. The lower limit of quantification was 2.0 ng/mL. A cross-validation analysis was performed using HPLC/MS/MS methodology (r = 0.88).

### Genotyping

Liver biopsies were performed on each patient as part of the study protocol. Liver biopsies were embedded in formaldehyde paraffin and subsequently stored. Sampling of paraffin-embedded tissue was performed; 5 slices of 10-μm-thick recipient liver biopsies were taken using a microtome. The extraction of genomic DNA from these samples was carried out using the commercial MagMAX™ FFPE DNA/RNA Ultra Kit (CAT A31881, Thermofisher), which is a separation system using magnetic beads. The protocol suggested in the insert was followed, with modifications in the deparaffinization time, going from 3 to 90 min, and in the tissue digestion process with protease, leaving it for 16 h. DNA concentration was measured using a NanoDrop spectrophotometer. To verify the quality and integrity of the extracted DNA, 1% agarose gels stained with RedGel were electrophoresed. The DNA was then stored at −80°C until the genotyping or sequencing process. CYP3A5 (CYP3A5*1/*3 or *3/*3) polymorphisms were assessed in liver biopsies (receptor tissue). CYP3A5*3 (rs776746) polymorphism was detected by PCR and directly sequenced. Patients who were carriers of this variant (CYP3A5*1/*1 or CYP3A5*1/*3) were selected first and were called ‘expresser’ patients. Recipients carrying the CYP3A5*3/*3 genotype, responsible for the lack of CYP3A5 expression, were selected second and called ‘non-expresser’ patients.

### Statistical analyses

Daily doses of TAC, Co, and Co/dose were estimated according to the expression or not of the CYP3A5*1 allele. All values were expressed as median and range or mean and confidence interval of 95%. To evaluate the longitudinal relationship between Co/doses, dose, and Co and genetic variables, a generalized estimating equations model was used using a log-gamma distribution, according to the recommendations of Chen et al., for modeling repeated concentration data (Chen and Lin, 2013). For permanence in the model, a significance level less than or equal to 5% was adopted as a criterion. All analyses were performed using the STATA 11.0^©^ program.

## Results

**FIGURE 1:**
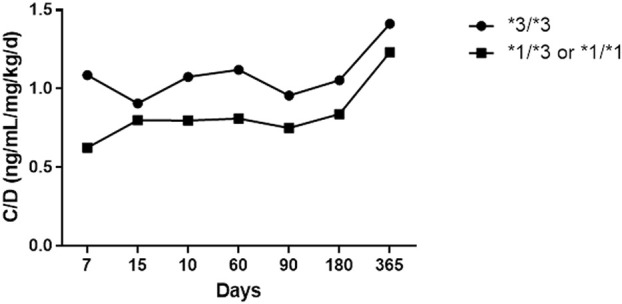
C0/dose ratio in expresser recipients over 2 years of follow-up.

We evaluated 16 patients who received TAC during the first 2 years after transplantation. Table 1 shows the characteristics of the population studied. We observed 12 patients (70%) with adverse events associated with tacrolimus, 5 patients (29%) had at least a rejection episode, and four patients died (40%) during follow-up. CYP3A5*1 expression was observed 28% of patients. There were no statistically significant deviations in the distribution of polymorphisms according to the Hardy-Weinberg principle (p > 0.05). Patients with CYP3A5 expressors displayed lower C0 and C0/dose ratio and higher doses than those no expressors, Table 1. We observed a lower C0/dose ratio in expresser recipients over 2 years of follow-up (Figure 1) (Table 2).

**TABLE 1:** Clinical profile of patients included.

Variable	
Male(%)	9 (52%)
Age (yr): Median (range)	50 (22–68)
Weight (kg): Median (range)	66,4 (49,1–100)
Primary disease	
Alcoholic cirrhosis	5 (29%)
primary sclerosing cholangitis	4 (23%)
Others^a^	12 (47%)
Transplant rejection confirmed by biopsy	5 (29%)
Length of stay (days)	18 (1–58)
Deaths	4 (40%)
Tacrolimus adverse events	12 (70%)
Neurotoxicity^a^	8 (47%)
Nephrotoxicity^b^	6 (35%)
CYP3A5	
CYP3A5^c^1/^c^1	4 (28%)
CYP3A5^c^3/^c^3	10 (71%)

^a^ Headaches, insomnia, tremor, paresthesia and dizziness.

^b^ Increased creatinine, azotemia, interstitial fibrosis, and oliguria.

^c^ Others: Autoimmune hepatitis, cryptogenic cirrhosis, acute liver failure.

**TABLE 2:** Pharmacokinetics profile by CYP3A5 genotype.

	CYP3A5*1/*1 median and 95% conf. interval	CYP3A5*3/*3 median and 95% conf. interval	p
Blood concentration (ng/mL)	6.85 (5.47–8.23)	8.65 (0 0.64 to 5.47)	0.031
Dose (mg/kg/day)	7.76 (6.59–8.92)	5.76 (4.55–6.97)	0.005
Concentration dosage ratio (ng/mL) (mg/kg/day)	1.04 (0.60–1.48)	1.46 (1.11–1.80)	0.003

## Discussion

Our research evidenced the differential impact over time of the presence of CYP3A5 polymorphisms on the pharmacokinetics of tacrolimus. Unlike most studies published with short follow-up periods and generally in a non-Hispanic population, this is the first study in the Colombian liver transplant population to evaluate the two-year effect of these polymorphisms.

Individuals possessing at least one A allele at CYP3A5 (rs776746), referred to as CYP3A5*1 or the wild type, have enhanced the enzymatic activity responsible for metabolizing tacrolimus (TAC) (Kuehl et al., 2001). The frequency of expressors (CYP3A5*1) in our study (28%) is at an intermediate value between that published in the Asian (33%–66%) and Caucasian (9%–15%) populations, and similar to what it was found in Argentina (Buendia et al., 2015; Buendia et al., 2014; Riva et al., 2018; Buendia et al., 2020; Lavandera et al., 2010; Larriba et al., 2010; Ferraris et al., 2011). These intermediate values, between the Caucasian and Asian frequency, can reveal the genetic diversity present in Latin America (Roco et al., 2012). These results highlight the importance and need for more pharmacogenetic studies in Latin American patients, which allow improving the understanding of the bases in ethnic variations in metabolism and the effect of different drugs; in this way not continue to extrapolate results obtained from other populations which lead or may lead to inaccurate estimates.

In patients following liver transplantation, both donor and recipient CYP3A5 polymorphisms are associated with changes in CT pharmacokinetics. In our study, we revealed that these pharmacokinetic differences, although more pronounced in the first months after transplantation, are maintained until 2 years of follow-up. In a previous study with a larger sample size in the Argentine population, we also demonstrated differential behavior according to the presence of CYP3A5 expression in the pediatric population (Buendia et al., 2020). These findings are consistent with a study conducted by Uesugi (Uesugi et al., 2014) in which he evaluated the impact of CYP3A5 in the first 5 weeks post-transplant in 410 adult liver transplants. This study reveals that expressing recipient patients presented significantly (p < 0.001) lower Co/dose, compared to non-expressing recipients, regardless of the genotype of the donor’s liver in the first weeks after transplantation. Even these differences are more marked in the first 2 weeks after transplantation (p = 0.035), as presented in the study of Wang (2014), in a study in 96 liver transplanted adults, after which the differences in Co/dose between expressing and non-expressing receptors are reduced, and lose statistical significance (p = 0.08) (Wang et al., 2014).

One of the strengths of our study is the rigorous selection of patients to minimize potential confounders affecting tacrolimus pharmacokinetics. Importantly, none of the patients in our cohort had documented consumption of grapefruit or grapefruit juice, which is known to inhibit CYP3A enzymes and alter tacrolimus metabolism. Additionally, no patients were prescribed erythromycin, fluoxetine, paroxetine, ketoconazole, itraconazole, azamulin, or verapamil, which are known inhibitors of tacrolimus metabolism. Similarly, none of the participants were taking rifampin, carbamazepine, or phenytoin, potent inducers of CYP3A5 that could have significantly affected tacrolimus clearance. The absence of these pharmacokinetic modifiers strengthens the validity of our findings by ensuring that the observed variability in tacrolimus exposure is primarily attributable to CYP3A5 polymorphisms rather than external pharmacological influences.

In our analysis of the four patients who died during follow-up, we did not observe significant differences in their tacrolimus pharmacokinetic parameters compared to the rest of the cohort. Their trough concentrations (C_0_), dose-adjusted concentrations (C_0_/dose ratio), and total tacrolimus doses remained within the expected ranges observed for the study population. This suggests that factors other than tacrolimus pharmacokinetics may have contributed to their clinical outcomes. However, given the small sample size, we recognize the need for larger studies to further explore potential pharmacokinetic differences in patients with poorer prognoses. Future research should aim to integrate clinical, genetic, and pharmacokinetic variables to better understand the multifactorial nature of outcomes in liver transplant recipients.

To our knowledge, this is the first study evaluating the impact of CYP3A5 polymorphisms on tacrolimus pharmacokinetics in Colombian liver transplant patients. Our findings contribute to the growing body of evidence on pharmacogenetic variability in immunosuppressive therapy, particularly in Latin American populations. However, this study has some limitations. It is an exploratory study with a small sample size, which may affect the statistical power of our findings. Despite this limitation, our results provide valuable preliminary data that can guide future confirmatory studies in our country. Additionally, we acknowledge the lack of information on donor genotypes, which could influence tacrolimus metabolism and pharmacokinetics. Previous studies have shown that both donor and recipient CYP3A5 polymorphisms may impact drug exposure, particularly in the early post-transplant period. Future research should aim to incorporate larger sample sizes and donor genetic data to better characterize the interaction between donor and recipient CYP3A5 expression and its clinical implications. In conclusion, in patients after liver transplantation, CYP3A5 polymorphisms of the recipient are associated with changes in CT pharmacokinetics. These changes, although marked in the first days after transplantation, remain in the first 2 years of follow-up.

## Data Availability

The raw data supporting the conclusions of this article will be made available by the authors, without undue reservation.
